# Abnormal Cerebellar Volume in Patients with Remitted Major Depression with Persistent Cognitive Deficits

**DOI:** 10.1007/s12311-020-01157-z

**Published:** 2020-07-08

**Authors:** Malte S. Depping, Mike M. Schmitgen, Claudia Bach, Lena Listunova, Johanna Kienzle, Katharina M. Kubera, Daniela Roesch-Ely, R. Christian Wolf

**Affiliations:** Center for Psychosocial Medicine, Department of General Psychiatry, University of Heidelberg, Vossstr. 4, 69115 Heidelberg, Germany

**Keywords:** Magnetic resonance imaging, Major depressive disorder, Cerebellum, Cerebellar gray matter, Attention, Executive function

## Abstract

Cerebellar involvement in major depressive disorder (MDD) has been demonstrated by a growing number of studies, but it is unknown whether cognitive functioning in depressed individuals is related to cerebellar gray matter volume (GMV) abnormalities. Impaired attention and executive dysfunction are characteristic cognitive deficits in MDD, and critically, they often persist despite remission of mood symptoms. In this study, we investigated cerebellar GMV in patients with remitted MDD (rMDD) that showed persistent cognitive impairment. We applied cerebellum-optimized voxel-based morphometry in 37 patients with rMDD and with cognitive deficits, in 12 patients with rMDD and without cognitive deficits, and in 36 healthy controls (HC). Compared with HC, rMDD patients with cognitive deficits had lower GMV in left area VIIA, crus II, and in vermal area VIIB. In patients with rMDD, regression analyses demonstrated significant associations between GMV reductions in both regions and impaired attention and executive dysfunction. Compared with HC, patients without cognitive deficits showed increased GMV in bilateral area VIIIB. This study supports cerebellar contributions to the cognitive dimension of MDD. The data also point towards cerebellar area VII as a potential target for non-invasive brain stimulation to treat cognitive deficits related to MDD.

**Electronic supplementary material:**

The online version of this article (10.1007/s12311-020-01157-z) contains supplementary material, which is available to authorized users.

## Introduction

Cognitive dysfunction is a highly relevant symptom domain in major depressive disorder (MDD), affecting about two-thirds of acutely depressed patients [1]. In patients with MDD, cognitive impairment independently mediates functional outcomes, e.g., workforce performance [2]. Cognitive dysfunction in depression typically includes deficits of attention and executive functions [1, 3]. Importantly, in 30–50% of patients with MDD, cognitive dysfunction persists despite remission of mood symptoms [1, 4]. If persistent in remitted individuals with MDD, such impairments may mediate an increased risk of major depression relapse [5–7]. Cognitive dysfunction in MDD is difficult to treat with little evidence for procognitive effects of antidepressant medication [8]. Non-pharmacological interventions, such as cognitive remediation therapy, may be effective, yet data in this regard is scarce and treatment benefits are poorly defined [9].

Against this background, there is an urgent need for a more detailed understanding of the neural mechanisms of cognitive dysfunction in MDD. Using structural and functional magnetic resonance imaging (MRI), neural correlates of cognitive dysfunction in depression have been identified by numerous studies, including meta-analyses [10–13]. Although the past decade has witnessed an increased interest in cerebellar involvement in depression, potential cerebellar contributions to cognitive dysfunction in MDD have rarely been investigated [14]. Since approximately half of the cerebellar cortex is associated with non-motor functions, i.e., cognitive, affective, and self-referential functions [15], this may be a serious omission.

Functional MRI (fMRI) analyses of cerebro-cerebellar connectivity in large human samples have revealed a clear cerebellar topography, i.e., parallel neural circuits that the cerebellar subregions form with different functional areas of the cerebral cortex and with different subcortical structures [15]. There is a special role for cerebellar area VII. Unlike all other cerebellar areas, area VII is not connected with the somatomotor system but exclusively communicates with cerebral structures that support cognitive, affective, and self-referential processes [16]. Additional non-motor representations are located in cerebellar areas VI and IX [16].

Investigations of cerebellar structure and function with MRI require optimized data analysis methods. Image registration should employ cerebellum-optimized templates, since the use of conventional templates results in anatomically imprecise findings [17, 18]. A small number of cerebellum-optimized MRI investigations in MDD have been published so far. Voxel-based morphometry (VBM) with cerebellum-optimized data analysis has been applied by two studies [19, 20]. Cerebellar resting perfusion has been examined by one fMRI study [21]. In patients with acute depression, abnormal gray matter volume (GMV) has been demonstrated in area IX, a region belonging to the so-called default-mode network (DMN) [20]. Additionally, in patients with acute MDD, abnormal resting perfusion within area VIIA has been associated with depressive symptom load [21]. However, neither of those studies specifically addressed cognitive functions. A few fMRI studies have investigated cerebellar intrinsic connectivity, yet cerebellum-optimized data analysis was not applied in any of those studies [14, 22–26]. Keeping this important limitation in mind, there is preliminary evidence for abnormal resting-state connectivity of area VIIA with cerebral components of the so-called cognitive control network (CCN) in patients with acute depression [14, 24, 25].

This study investigated structural cerebellar correlates of persistent cognitive dysfunction in patients with remitted depression. Cognitive functioning in mental disorders can be conceptualized as a dimensional construct [27]. Along a spectrum of functioning, this study included depressed patients with a more severe impairment of attention and executive functions. The study aimed at cerebellar mechanisms that contribute to the cognitive dimension of MDD. The study subjects were participants in a project that evaluated cognitive remediation therapy in remitted MDD [28]. For this study, patients were investigated at baseline, i.e., prior to cognitive training. Based on evidence for cognitive processes predominantly subserved by area VII, and based on cumulative data suggesting abnormal structure and function of this cerebellar subregion in acute depression, we hypothesized that patients with remitted depression and persistent cognitive deficits would show abnormal gray matter volume (GMV) of area VII. We also expected that area VII GMV in patients would be significantly associated with cognitive performance, as measured by a comprehensive neuropsychological test battery.

## Material and Methods

### Patient Characteristics

This study was part of a project that examined the effects of cognitive training in patients with remitted MDD. Patient recruitment and selection have been described in detail elsewhere, as have been the preliminary behavioral outcomes of that project [28]. Briefly, patients were diagnosed at the Department of General Psychiatry, University Hospital Heidelberg, using the Structured Clinical Interview for DSM-IV (SCID-I). Patients with a history of MDD and remission at the time of the examination were included in the study. Four neuropsychological domains were evaluated: attention, processing speed, executive functions, and learning/memory, for details see section “Neuropsychological Evaluation.” Patients were considered to be cognitively impaired, if they showed deficits (standardized test values of *z* < − 1) in at least two out of six cognitive subdomains (alertness, selective attention, divided attention, working memory, inhibition, planning; for details see section “Neuropsychological Evaluation”). Only subjects between 18 and 60 years of age and with an IQ > 80 according to the Multiple Choice Vocabulary Test (MWT-B, [29]) were included. Exclusion criteria were comorbid psychiatric disorders according to DSM-IV (except dysthymia), neurological disease, or past traumatic brain injury. MRI examinations were performed prior to cognitive remediation training. MRI examinations were carried out in 37 patients with remitted MDD and persistent cognitive dysfunction (rMDD with c.d.), in 12 patients with remitted MDD and without cognitive deficits (rMDD without c.d.), and in 36 healthy volunteers (HC), see Table 1. The median estimated duration of current remission—according to the patient’s assessment—was 7.6 months in patients with persistent cognitive deficits and 8.6 months in patients without cognitive deficits (*p* = 0.56). At the time of study enrollment, 47 patients were treated as outpatients, and 2 patients were treated at a psychiatric day hospital. Any psychotropic medication established in patients’ regular treatment remained unchanged. Of all patients with persistent cognitive deficits, 20 received psychotropic medication (venlafaxine = 5, escitalopram = 3, citalopram = 3, vortioxetine = 2, agomelatine = 1, amitriptyline = 1, bupropion = 1, duloxetine = 1, fluvoxamine = 1, quetiapine = 1, sertraline = 1). Of all patients without cognitive deficits, 5 received psychotropic medication (sertraline = 2, citalopram = 1, fluoxetine = 1, venlafaxine = 1). Of all patients receiving psychotropic medication, all but two patients were on a monotherapy. No patient had ever received electroconvulsive therapy. All control subjects were medication-free (except for contraceptives). All subjects were right-handed. The study was approved by the local ethics committee (Heidelberg University) and carried out in accordance with the Helsinki Declaration. Written informed consent was obtained from all participants.

**Table 1:** Demographics and clinical variables for patients with remitted major depression (rMDD) with or without persistent cognitive deficits (c.d.), as well as for healthy controls (HC). *HAMD*, Hamilton Depression Rating Scale; *sd*, standard deviation; *n.a.*, not applicable

	rMDD with c.d. (*n* = 37)		rMDD without c.d. (*n* = 12)		HC (*n* = 36)		
	Mean	sd	Mean	sd	Mean	sd	*p* value
Age (years)	45.0	12.7	40.6	12.8	40.5	12.2	0.276^a^
Gender (m/f)	15/22	n.a.	1/11	n.a.	12/24	n.a.	0.121^b^
Education (years)	11.9	1.7	12.3	0.9	11.7	1.5	0.464^a^
Duration of illness (years)	14.5	12.9	15.6	11.9	n.a.	n.a.	0.803^c^
Number of episodes	4.2	7.1	3.5	3.2	n.a.	n.a.	0.752^c^
HAMD	4.9	3.6	5.8	4.1	0.4	0.8	< 0.001^a^

^a^ANOVA

^b^Chi-square test

^*c*^*t* test

### Neuropsychological Evaluation

Four cognitive domains were tested: (1) Attention was assessed using the Perception and Attention Functions Battery (WAF). Three different attention domains were tested, i.e., alertness (WAFA, performance measure [PM]: response time), selective attention (WAFS, PM: number of errors), and divided attention (WAFG, PM: number of omissions). (2) Processing speed was assessed using the Trail Making Test-A (TMT-A, PM: completion time) and the Digit Symbol Substitution Test (DSST, PM: number of correct assignments). (3) Assessment of executive functions included tests to evaluate working memory (N-Back verbal, PM: number of errors), inhibition (Go-Nogo, PM: response time, number of errors), cognitive flexibility (Trail Making Test-B, PM: completion time), and planning (Tower of London (ToL), PM: number of correctly solved tasks). (4) Assessment of learning and memory included tests for verbal memory (California Verbal Learning Test (CVLT), PM: number of words recalled, immediate and delayed recall accuracy) and figural memory (Figural Memory Test, PM: number of figures recalled, immediate and delayed recall accuracy). The neuropsychological tests were included in the computerized “Vienna Test System” (Schuhfried GmbH, 2012, http://www.schuhfried.at). Raw test values were *z*-transformed and polarized into one direction with higher *z*-values indicating better cognitive performance. Individual test scores were averaged to form four domain scores ​(attention, processing speed, executive functions, learning, and memory), see Table 2. Domain scores were additionally averaged to form a general cognitive composite score, see Table 2.

**Table 2:** Cognitive performance in patients with remitted major depression (rMDD) with or without persistent cognitive deficits (c.d). Raw values from single neuropsychological tests were *z*-transformed and averaged to form composite scores for four cognitive domains, see the “Material and Methods” section for details. In addition, a general composite score was formed; see the “Material and Methods” section for details. The table shows *z*-values. *sd*, standard deviation

	rMDD with c.d. (*n* = 37)		rMDD without c.d. (*n* = 12)		
	Mean	sd	Mean	sd	*p* value
Attention	− 0.24	0.54	0.46	0.52	< 0.001^a^
Processing speed	− 0.25	0.94	0.38	0.64	0.036^a^
Learning and memory	− 0.12	0.73	0.07	0.82	0.445^a^
Executive function	− 0.25	0.61	0.27	0.36	0.008^a^
General composite score	− 0.22	0.55	0.30	0.49	0.006^a^

^a^*t* test

### MRI Data Acquisition

MRI scans were acquired using a 3 Tesla Siemens Magnetom TIM Trio scanner, located at the Department of Neuroradiology, University Hospital Heidelberg. T1-MPRAGE sequences were acquired with the following parameters: TE = 2.52 ms; TR = 1900 ms; TI = 900 ms; FOV = 256 mm; slice thickness = 1 mm; resolution = 1.0 × 1.0 × 1.0 mm; number of slices = 256.

### MRI Data Analysis

The Statistical Parametric Mapping Software, Version 12 (SPM 12, http://www.fil.ion.ucl.ac.uk/spm), implemented in MATLAB R2019a, was used to analyze MRI data. The Spatially Unbiased Infratentorial Toolbox (SUIT, http://www.diedrichsenlab.org/ima-ging/suit.htm) was used for cerebellum-optimized VBM. Individual T1-weighted sequences were controlled for scanner artifacts and the image origin was set at the anterior commissure. The infratentorial structures, i.e., cerebellum and brain stem, were isolated from the surrounding tissue. Using the unified segmentation approach [30], the infratentorial structures were segmented into gray matter (GM), white matter, and cerebrospinal fluid. Using the Diffeomorphic Anatomical Registration Through Exponentiated Lie Algebra (DARTEL) algorithm [31], the individual GM segments were registered onto the SUIT atlas template. In order to preserve individual anatomical differences, the GM probability maps were modulated using the deformation fields that resulted from the registration procedure, providing GMV maps [32]. The GMV maps were smoothed with a Gaussian kernel of 4-mm full width at half-maximum (FWHM) [33].

**Fig. 1:**
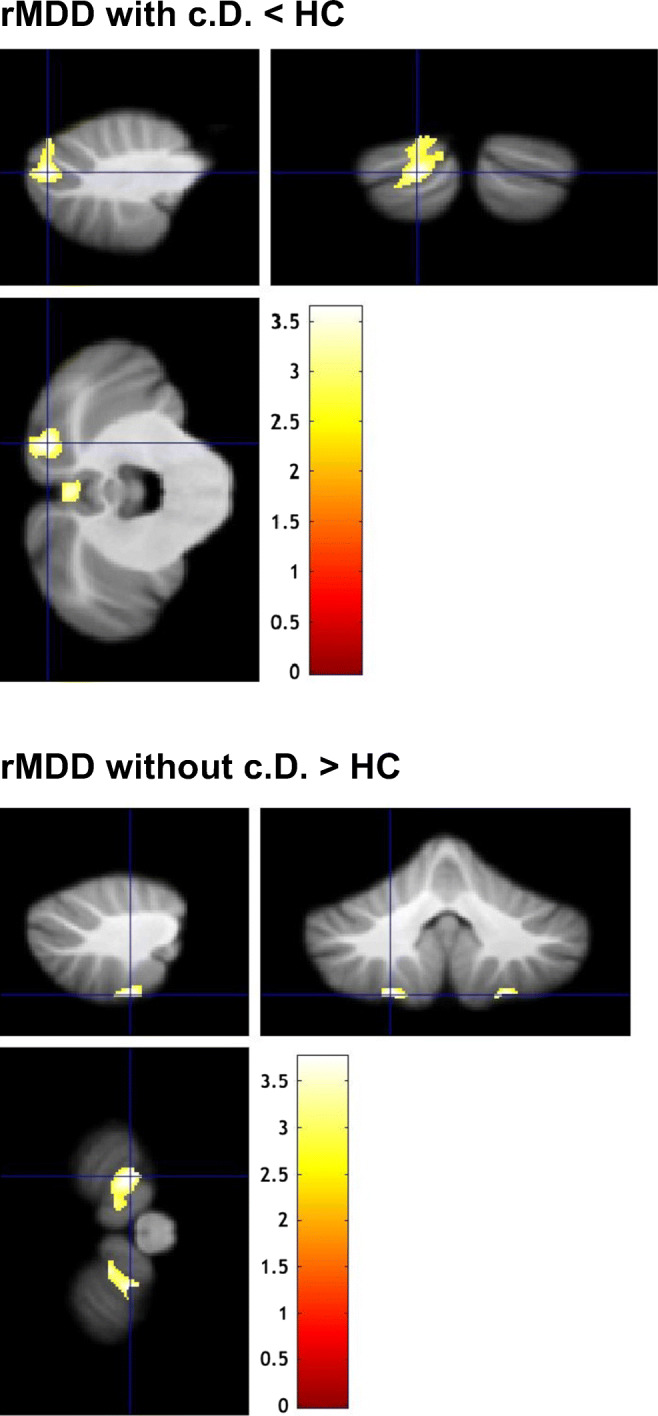
Cerebellar regions showing aberrant gray matter volume in patients with remitted MDD (rMDD) with or without cognitive deficits (c.d.), each compared with healthy controls (HC). Sagittal, coronal, and horizontal views (clockwise, starting from top left). Results of second-level between-group analyses (two-sample *t* tests; nuisance variables: age, gender, years of education, and depression severity, i.e., HAMD-Score), *p* < 0.005 uncorrected for height, corrected for spatial extent using an empirically determined threshold of *k* > 259 voxels, see the “Material and Methods” section for details. The color bar represents *t* values

Voxel-wise statistical analyses were applied to assess the effect of cognitive impairment on regional cerebellar GMV. We used the general linear model (GLM) approach, as implemented in SPM 12, to investigate the effect of group (remitted MDD with cognitive deficits, remitted MDD without cognitive deficits, HC) upon voxel-wise GMV, resulting in voxel-wise parameter estimates. Age, gender, years of education, and depression severity (i.e., HAMD score [34]) were included as nuisance variables. For completeness, an additional GLM was calculated, where depression severity was not included as a nuisance variable. To investigate between-group differences, voxel-wise *t* tests were calculated. As in previous studies [35, 36], given a strong a priori hypothesis, we chose a significance threshold of *p* < 0.005 (uncorrected at the voxel level) to assess between-group differences. Based on random field theory [37], an empirically determined extent threshold according to the expected number of voxels per cluster within the respective contrast was applied. Anatomical localizations were determined using a probabilistic MRI atlas of the cerebellum [38], as implemented in the SPM Anatomy Toolbox [39]. For a graphical summary of results, color-coded statistical maps were created, see Fig. 1.

### Correlations with Clinical and Neuropsychological Scores

Within the patient group, explorative correlation analyses were calculated between regional morphometric effects and clinical variables (duration of illness and number of depressive episodes), as well as cognitive parameters (four cognitive domain scores and general cognitive composite score). To this end, the first eigenvariate of the parameter estimates was extracted from cerebellar clusters showing between-group differences. Correlations were calculated using the Prism 6 software (http://www.graphpad.com/scientific-software/prism). A nominal threshold of *p* < 0.05 was defined (uncorrected for multiple comparisons).

## Results

Compared with HC, patients with remitted MDD and persistent cognitive deficits (rMDD with c.d.) showed a GMV reduction within left area VIIA, crus II, and vermal area VIIB (see Fig. 1 and Table 3). Patients with remitted MDD and without cognitive deficits (rMDD without c.d.) showed a GMV increase within bilateral area VIIIB compared with HC (see Fig. 1 and Table 3). No significant GMV differences were detected between the two patient groups.

**Table 3:** Cerebellar regions showing aberrant gray matter volume in patients with remitted major depression (rMDD) with or without cognitive deficits (c.d.), each compared with healthy controls (HC). The table shows *t* values and stereotaxic coordinates (*x*, *y*, *z*) for peak voxels emerging from second-level between-group comparisons, see the “Material and Methods” section for details. Peak voxel coordinates were assigned to probabilistic cytoarchitectonic maps, providing the probability and the 95% confidence range of belonging to a specific cerebellar area. *SUIT*, Spatially Unbiased Infratentorial Toolbox

	SUIT analysis					
	Cerebellar region	*x*	*y*	*z*	*t*-value	No. of voxels
rMDD with c.d. < HC	Left area VIIA, crus II (69%, 12–69%) *Left area VIIA, crus I* *(31%, 31–84%)*	− 17	− 83	− 34	3.62	1128
	Vermal area VIIB (56%, 11–56%)	1	− 74	− 34	3.40	451
rMDD without c.d. > HC	Left area VIIIb (92%, 83–95%)	− 21	− 51	− 60	3.76	444
	Right area VIIIb (96%, 76–96%)	22	− 52	− 60	3.75	282

The cerebellar region printed in italics belongs to the voxel coordinates provided in the row above, i.e. there is a 69% probability that x=-17, y= -83, z= -24 belongs to left area VIIA, crus II (top row), and a 31% probability that x= -17, y= -83, z= -24 belongs to left area VIIA, crus I (lower row)

In MDD patients, significant correlations were found between GMV of the cluster in left area VIIA, crus II, and attention functioning (*r* = 0.50, *p* < 0.001), executive performance (*r* = 0.38, *p* = 0.007), and general cognitive functioning, i.e., the cognitive composite score (*r* = 0.36, *p* = 0.012). Additionally, significant correlations were found between GMV of the cluster in vermal area VIIB and attention functioning (*r* = 0.44, *p* = 0.001), executive performance (*r* = 0.42, *p* = 0.003), and general cognitive functioning (*r* = 0.36, *p* = 0.012). There were no significant correlations between GMV and clinical variables, i.e., duration of illness or number of depressive episodes. When correcting the correlation analyses for multiple comparisons using the Bonferroni method (*p* < 0.0071), correlations between GMV in both clusters and attention and executive performance, respectively, remained significant, while correlations between GMV in both clusters and general cognitive functioning showed a trend towards significance.

To test for potential effects of depression severity, we excluded the HAMD score as a nuisance variable in the first-level analysis and recalculated all second-level analyses. This approach did not change our main findings, except for the cluster in vermal area VIIB in patients with cognitive deficits (see Supplementary Material, Table 1).

## Discussion

This study investigated cerebellar GMV in patients with remitted MDD with or without cognitive deficits compared with healthy controls. Two main findings emerged: First, patients with remitted MDD and cognitive deficits showed reduced GMV within area VIIA, crus II, and vermal area VIIB, which was associated with attention deficits and impaired executive performance. Second, patients with remitted MDD without cognitive deficits showed increased GMV in area VIIIB.

So far, cognitive deficits in depression have mainly been associated with structural and functional changes of cerebral regions [10–13]. In line with our hypothesis, this study revealed abnormal structure of cerebellar area VII in patients with remitted depression, which was linked to cognitive dysfunction. This adds to a recent body of research that consistently demonstrated contributions of area VII to cognitive functioning in MDD [16]. Indeed, cerebellar contributions to cognitive dysfunction in MDD are now better documented than potential cerebellar correlates of abnormal affective and self-referential processing [16].

Our data are in agreement with the notion that recurrence risk in depression may be related to state-independent abnormalities of brain structure and function [40]. Such neural abnormalities may accumulate with increasing number of depression relapses and they may in turn convey an increased risk for relapse [41–43]. Cognitive dysfunction is considered to be one of the clinical mediators of depression relapse [5–7]. In line with this, this study shows cerebellar GMV changes in remitted depression and an association of abnormal cerebellar GMV with cognitive dysfunction. Future longitudinal studies should specifically address whether cerebellar abnormalities persisting into remission may be associated with risk of MDD relapse.

It is important to emphasize that performance deficits that can be assessed in neuropsychological tests represent only one facet of cognitive dysfunction in depression. At the core of cognitive depression theories, cognitive dysfunction is being related to preferential processing of negative emotional information and dysfunctional emotion regulation, e.g. rumination [44, 45]. Future studies should investigate potential cerebellar contributions to these depression-specific cognitive processes. Schmahmann and colleagues have suggested that cerebellar dysfunction may lead to “cognitive-affective dysmetria”, i.e., to specific abnormalities of cognitive and affective processes, in analogy to the well-defined “motor dysmetria” in cerebellar disorders [46]. This is a highly attractive model, but empirical support in patients with MDD is lacking at present. So far, no studies have investigated the mechanisms by which cerebellar abnormalities in MDD may be related to negative cognitive styles or impaired cognitive control. At this point, such cognitive abnormalities have only been associated with dysfunction of the cerebral components of the so-called cognitive control network [47, 48]. In future studies, potential associations between depressive cognition and abnormal cerebellar function or abnormal cerebro-cerebellar functional connectivity should be of particular interest [22]. Such investigations will help to validate and refine the model of “cognitive-affective dysmetria” as a result of cerebellar dysfunction.

In MDD treatment, non-invasive regional brain stimulation, e.g., transcranial magnetic stimulation (TMS), promises to reduce depressive symptoms by modulating neural activity in brain regions associated with cognitive processes [49]. TMS is typically applied over the lateral frontal cortex [50]. In light of our findings, cerebellar area VII should receive further attention as a possible target for TMS. In fact, pilot studies that applied TMS to the cerebellum are available [51–53].

In this study, patients with remitted MDD that showed intact cognitive functioning displayed increased volume of cerebellar area VIIIB compared with controls. Area VIIIB is associated with sensorimotor functions [54]. Psychomotor retardation is a major feature of MDD [55], but its neural correlates have been addressed by only a few studies [56, 57]. We have previously detected abnormal resting state perfusion in area VIIIB in acutely depressed patients [21]. We did not systematically assess psychomotor symptoms in this study (nor in our previous study), so we cannot make inferences about a potential psychomotor impact of abnormal area VIIIB structure (or function) in MDD. Interestingly, in patients with acute major depression, we previously found increased GMV in different cerebellar subregions [19, 20]. The causes of increased cerebellar GMV in MDD are unknown at this point of research. However, given that cerebellar plasticity is well recognized, it may be speculated that increased cerebellar GMV in depression may reflect compensation efforts, i.e., the so-called cerebellar reserve [58].

We acknowledge potential limitations of our study. Psychopharmacological treatment in patients was heterogeneous. Medication effects on cerebellar structure cannot be excluded. Also, more female than male subjects participated in this study. The disproportion was greatest in the subgroup of MDD patients without cognitive deficits. To correct for possible gender effects, gender was included as a nuisance variable in the statistical analysis of morphometric effects. Neuropsychological evaluation was not performed in healthy controls, preventing cerebellar structure—cognition analyses in those subjects. No patients with treatment-resistant depression were investigated in this study; thus, no claims can be made about abnormal cerebellar structure in such an MDD subgroup. Also, this study did not include patients with geriatric depression, where depressive symptoms and cognitive dysfunction may arise from accelerated brain aging. Thus, age-related cerebellar abnormalities in MDD cannot be addressed. Eventually, due to the cross-sectional study design, no conclusions can be made about the temporal stability of the cerebellar GMV findings.

## Conclusion

Keeping potential shortcomings in mind, this study demonstrated that MDD patients with current depressive symptom remission had abnormal GMV in both hemispheric and vermal portions of cerebellar area VII, which was associated with deficits of attention and executive function. The data support relevant contributions of the cerebellum to the cognitive dimension of MDD. Future studies should address whether abnormal area VII structure is associated with increased risk of depression relapse, whether abnormal area VII structure is linked to abnormal cognitive processes in depression (such as preferential processing of negative information or dysfunctional explicit emotion regulation), and whether abnormal area VII structure is associated with abnormal cerebellar activity or with abnormal cerebro-cerebellar functional connectivity. Future studies will particularly benefit from investigating MDD patients along a spectrum of cognitive functioning, as promoted by the Research Domain Criteria Initiative (RDoC) [27]. In MDD research, there has been an increasing interest in neural abnormalities at the functional network level [59]. In this context, computational approaches hold promise for identification of clinically useful biomarkers [60, 61]. Our results suggest that the cerebellum should be included in future analyses of neural networks in MDD, particularly when the cognitive dimension of depression is investigated. Finally, our results indicate that cerebellar area VII should be evaluated as a target for brain stimulation to treat cognitive deficits related to MDD.

## Electronic Supplementary Material

ESM 1 (DOC 34 kb).
