# Correction to: Abnormal Cerebellar Volume in Patients with Remitted Major Depression with Persistent Cognitive Deficits

**DOI:** 10.1007/s12311-021-01294-z

**Published:** 2021-06-19

**Authors:** Malte S. Depping, Mike M. Schmitgen, Claudia Bach, Lena Listunova, Johanna Kienzle, Katharina M. Kubera, Daniela Roesch-Ely, R. Christian Wolf

**Affiliations:** Center for Psychosocial Medicine, Department of General Psychiatry, University of Heidelberg, Vossstr. 4, 69115 Heidelberg, Germany

**Correction to: The Cerebellum (2020) 19:762–770**

10.1007/s12311-020-01157-z

The article “Abnormal Cerebellar Volume in Patients with Remitted Major Depression with Persistent Cognitive Deficits”, written by Depping et al, was originally published Online First without Open Access. After publication in volume 19, issue 6, page 762–770 the author decided to opt for Open Choice and to make the article an Open Access publication.

Therefore, the copyright of the article has been changed to © The Author(s) 2021 and the article is forthwith distributed under the terms of the Creative Commons Attribution 4.0 International License, which permits use, sharing, adaptation, distribution and reproduction in any medium or format, as long as you give appropriate credit to the original author(s) and the source, provide a link to the Creative Commons licence, and indicate if changes were made. The images or other third party material in this article are included in the article's Creative Commons licence, unless indicated otherwise in a credit line to the material. If material is not included in the article's Creative Commons licence and your intended use is not permitted by statutory regulation or exceeds the permitted use, you will need to obtain permission directly from the copyright holder. To view a copy of this licence, visit http://creativecommons.org/licenses/by/4.0/.

