# Supplementary material for: Abnormal Cerebellar Volume in Patients with Remitted Major Depression with Persistent Cognitive Deficits
Source: Cerebellum. 2020 Jul 8;19(6):762–70. doi: 10.1007/s12311-020-01157-z (PMC8214579; doi:10.1007/s12311-020-01157-z)
Supplement: Supplementary file 1 — (DOC 34 kb). [file 12311_2020_1157_MOESM1_ESM.doc]

|  | **SUIT analysis** | | | | |  |  |
| --- | --- | --- | --- | --- | --- | --- | --- |
|  | cerebellar region | x | y | z | t-value | no. of voxels |  |
| **rMDD with c.d. < HC** | left area VIIA, crus I  (82%, 31-82%)  *left area VIIA, crus II*  *(16%, 16-69%)* | -18 | -83 | -33 | 3.68 | 1656 |  |
| **rMDD without c.d. > HC** | left area VIIIb  (95%, 94-95%) | -18 | -53 | -60 | 3.69 | 423 |  |
|  | right area VIIIb  (96%, 94-100%) | 21 | -52 | -60 | 3.71 | 309 |  |

**Supplementary Material, Table 1. Cerebellar regions showing aberrant gray matter volume in patients with remitted major depression (rMDD) with or without cognitive deficits (c.d.), each compared to healthy controls (HC).** In this analysis, depression scores (HAMD) were not considered as nuisance variable. The table shows t-values and stereotaxic coordinates (x, y, z) for peak voxels emerging from 2^nd^ level between-group comparisons. Peak voxel coordinates were assigned to probabilistic cytoarchitectonic maps, providing the probability and the 95%-confidence range of belonging to a specific cerebellar area. SUIT: Spatially Unbiased Infratentorial Toolbox.
